# Cardiorespiratory Responses to 10 Weeks of Exoskeleton-Assisted Overground Walking Training in Chronic Nonambulatory Patients with Spinal Cord Injury

**DOI:** 10.3390/s21155022

**Published:** 2021-07-24

**Authors:** Jae Hyeon Park, Hyeon Seong Kim, Seong Ho Jang, Dong Jin Hyun, Sang In Park, JuYoung Yoon, Hyunseop Lim, Mi Jung Kim

**Affiliations:** 1 Department of Rehabilitation Medicine, Hanyang University Guri Hospital, 153 Gyeongchun-ro, Guri-si 11923, Korea; jhpark3.md@gmail.com (J.H.P.); systole77@hanmail.net (S.H.J.); 2 Department of Rehabilitation Medicine, Hanyang University College of Medicine, 222-1 Wangsimni-ro, Seoul 04763, Korea; khsyaa@daum.net; 3 Robotics Lab, R&D Division of Hyundai Motor Company, 37 Cheoldobangmulgwan-ro, Uiwang-si 16082, Korea; mecjin@gmail.com (D.J.H.); ppaksang@gmail.com (S.I.P.); maxfect@gmail.com (J.Y.); hyunseop@hyundai.com (H.L.)

**Keywords:** spinal cord injury, exoskeleton, walking, oxygen consumption, heart rate

## Abstract

Exercise intensity of exoskeleton-assisted walking in patients with spinal cord injury (SCI) has been reported as moderate. However, the cardiorespiratory responses to long-term exoskeleton-assisted walking have not been sufficiently investigated. We investigated the cardiorespiratory responses to 10 weeks of exoskeleton-assisted walking training in patients with SCI. Chronic nonambulatory patients with SCI were recruited from an outpatient clinic. Walking training with an exoskeleton was conducted three times per week for 10 weeks. Oxygen consumption and heart rate (HR) were measured during a 6-min walking test at pre-, mid-, and post-training. Exercise intensity was determined according to the metabolic equivalent of tasks (METs) for SCI and HR relative to the HR reserve (%HRR). Walking efficiency was calculated as oxygen consumption divided by walking speed. The exercise intensity according to the METs (both peak and average) corresponded to moderate physical activity and did not change after training. The %HRR demonstrated a moderate (peak %HRR) and light (average %HRR) exercise intensity level, and the average %HRR significantly decreased at post-training compared with mid-training (31.6 ± 8.9% to 24.3 ± 7.3%, *p* = 0.013). Walking efficiency progressively improved after training. Walking with an exoskeleton for 10 weeks may affect the cardiorespiratory system in chronic patients with SCI.

## 1. Introduction

Spinal cord injury (SCI) occurs in 0.93 million persons per year worldwide, and the age-standardized incidence rate is 13 per 100,000 people, causing a significant global injury burden [1]. Patients with SCI suffer from various health conditions, including pain, pressure ulcers, depression, bladder and bowel dysfunctions, impaired proprioception, and motor weakness [2,3]. Patients with SCI often have difficulty in walking due to motor weakness or impaired proprioception, which can lead to significant reductions in physical activities and a sedentary lifestyle [3,4]. Consequently, individuals with SCI have an increased risk of cardiovascular diseases [2,4]. Moreover, cardiovascular diseases have become a major cause of mortality in individuals with chronic SCI [5]. Therefore, lifestyle interventions have become an important issue in reducing cardiovascular mortality in patients with SCI [6].

Physical activity is an approved lifestyle intervention for cardiovascular fitness in patients with chronic SCI [4,6,7]. Exercise guidelines recommend at least 30 min of moderate to vigorous aerobic exercise three times a week to maintain cardiometabolic health [7,8]. Arm crank or wheelchair sports, such as wheelchair basketball or tennis, are common aerobic exercises for patients with chronic SCI who are wheelchair-dependent [4]. However, many individuals with SCI do not sufficiently participate in these exercises [9]. Furthermore, all these exercises are performed in a sitting position.

Robotic exoskeletons have been developed for individuals with difficulty in ambulation as an assistive gait training device and for personal use in daily activities [10,11]. Robotic exoskeletons provide external body weight support and lower extremity propulsion, which help non-ambulatory patients to walk. Furthermore, there has been growing interest in the use of robotic exoskeletons as an exercise modality. Walking training with a robotic exoskeleton is performed in a standing position, which has been reported to have many health and psychological benefits such as improved bowel function, blood circulation, quality of life, and better self-image [12]. Additionally, it has been reported that walking with an exoskeleton can elicit activation of trunk muscles below the level of injury, implying that more muscles may be recruited during exoskeleton-assisted walking than activities performed in sitting position [13]. Previous studies have demonstrated that, according to cardiorespiratory responses, the exercise intensity of walking with a robotic exoskeleton is moderate in patients with SCI despite different training sessions performed before measurements [14,15,16,17,18,19]. Although long-term physical activity with a moderate intensity level is known to induce positive cardiorespiratory responses, most studies have not reported changes in cardiorespiratory responses after exoskeleton-assisted walking training with improved proficiency in exoskeleton use. Only a few case studies have reported the changes in cardiorespiratory responses to six weeks of exoskeleton-assisted walking training [18,20]. However, these case studies reported inconsistent results and could not provide definite conclusions on the cardiorespiratory changes that occur in response to long-term exoskeleton use.

To date, the knowledge of the changes in cardiorespiratory responses to robotic exoskeleton-assisted overground walking training is limited. Thus, the purpose of this study was to investigate the exercise intensity of overground walking training with a robotic exoskeleton and to assess the changes in cardiorespiratory responses to robotic exoskeleton-assisted overground walking training in chronic nonambulatory patients with SCI.

## 2. Materials and Methods

### 2.1. Trial Design and Participants

This was a single-center, prospective, single-group observational study. Nonambulatory participants with SCI were recruited from an outpatient clinic in the rehabilitation department of a tertiary hospital. As this study is part of an early feasibility study for a newly developed exoskeleton, the sample size was determined to be 10 participants [21,22]. Functional improvement after exoskeleton-assisted walking has recently been published [21]. In this study, we focused on changes in cardiorespiratory responses after exoskeleton-assisted walking training. The inclusion criteria were (1) neurologically stable SCI without a change of motor or sensory level at least two months since injury, (2) age > 18 years, (3) body weight < 110 kg, (4) height between 1.6 and 1.8 m, (5) sufficient postural stability to perform a level transfer, and (6) sufficient upper-extremity strength to use a walker or crutch. The exclusion criteria were (1) spinal instability, (2) severe joint contracture in the lower extremities, (3) unhealed fracture in the lower extremities, (4) skin injuries in areas of contact with the device, (5) unresolved deep vein thrombosis, (6) uncontrolled hypertension or hypotension, (7) severe osteoporosis or osteoporotic fracture that could interfere with gait training, (8) spasticity of the lower extremities exceeding 3 out of 4 on the modified Ashworth scale, (9) functional limitation in the upper extremities due to weakness or contracture, (10) psychological or cognitive problems that may limit the ability to understand the investigator’s instructions, and (11) any other issue that may interfere with the trial.

### 2.2. Exoskeleton and Training

The Hyundai Medical Exoskeleton (H-MEX; Hyundai Motor Company, Uiwang-si, Korea) was used in this study. A detailed description of the H-MEX has been previously reported [21,23]. H-MEX weighs approximately 19 kg, generates walking propulsion via actuators for flexion/extension in the hip and knee joints, and provides locomotive stability by way of hip abduction during hip flexion and hip adduction during hip extension via abduction/adduction actuators for the pelvic joints. Locomotive stability was also achieved by using a ground reaction force sensor-based permission operation. The exoskeleton was adjusted to each participant’s length of the lower extremities (thigh and shank) and position of the joints (hip, knee, and ankle) before training. Walking was initiated and stopped using the controller in the crutch hand grip. The bipedal gait was initiated by pressing the button on the left crutch with unilateral forward movement and stopped by pressing another button on the left crutch.

Before the walking training, the participants were provided with a detailed explanation of how to handle the exoskeleton and underwent a pre-training session to adapt to the use of the exoskeleton. The training program was performed for a total of 30 sessions, with a 60-min duration, three times a week for 10 weeks. The training program consisted of sit-to-stand, walking, and stand-to-sit movements. The participants walked along a flat 20-m corridor under the researchers’ close supervision. At the end of the corridor, the turn was made by changing direction little by little with the assistance of the researcher. The participants were allowed to rest in a sitting position during the walking training session, as needed. During the training, an overhead harness system (LG-1000; Neotech Inc., Gimpo-si, Korea) was loosely connected to the exoskeleton without weight support to prevent falls. As the training progressed, the walking distance and walking time without rest gradually increased, and the researchers’ level of assistance gradually decreased.

### 2.3. Measurements

#### 2.3.1. Six-Minute Walking Test (6MWT)

Walking ability was assessed using the 6MWT at the pre-, mid- (at week 5, after 15 training sessions), and post-training time (after 30 training sessions) points during the exoskeleton training [24]. The initial 6MWT distance was measured after one to three acclimating sessions before the walking training began to allow the participants to become accustomed to the exoskeleton. Before the 6MWT, the participants were asked to rest in a sitting position for more than 5 min. The participants ambulated along a 20 m straight walkway, which was used as a training path. The 6MWT began with the first step. The participants made a turn at the end of the corridor. Resting was allowed during the 6MWT. The number and duration of rests were not recorded. The time taken to turn around at the end of the corridor and the resting time were both included in the 6MWT. If the participants wanted to discontinue walking, the test was terminated and the distance at that time was recorded. Walking speed was determined by dividing the total walking distance by six minutes (m/min).

#### 2.3.2. Cardiorespiratory Measurements

**Figure 1:**
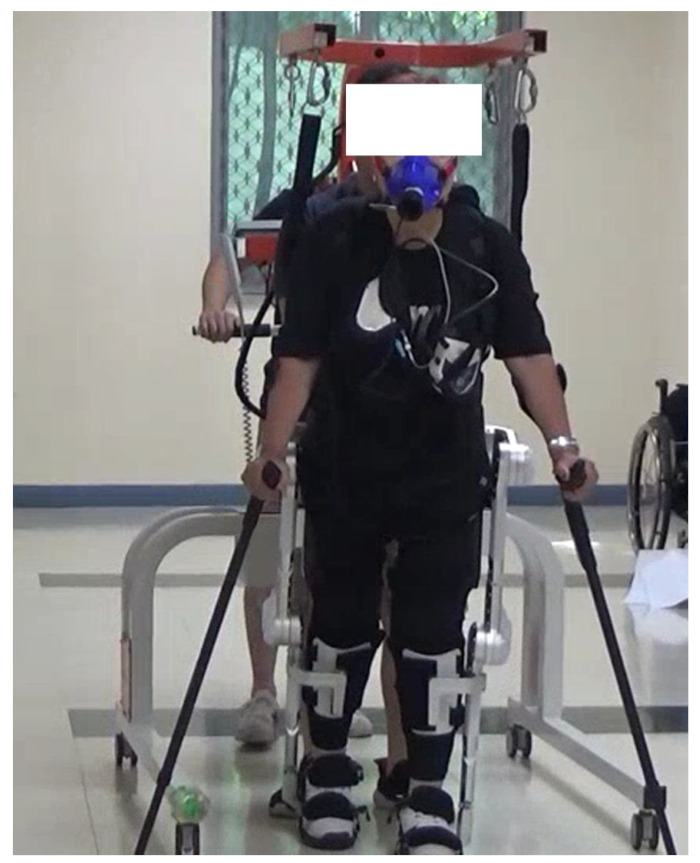
Exoskeleton-assisted overground walking with a portable gas analyzer.

Cardiorespiratory assessments were performed at pre-, mid-, and post-training time with the 6MWT. Cardiorespiratory measurements were obtained using the Metamax 3B portable gas analysis system (Cortex Medical, Leipzig, Germany) (Figure 1), which has been shown to be reliable and valid [25]. Prior to testing, calibration was performed according to the manufacturer’s guidelines. The respiratory measurements and heart rates for the breath-by-breath data were taken every 3 s. Cardiorespiratory measurements included volume of oxygen consumption (VO_2_, mL/kg/min) and heart rate (HR, beats/min). VO_2_ was determined using standard metabolic algorithms, and HR was measured using a polar belt. The data were analyzed using Metasoft 3 software. Before the 6MWT, VO_2_ during rest (VO_2rest_) and HR during rest (HR_rest_) were measured while wearing the exoskeleton during a 1-min of resting period. The highest VO_2_ (VO_2peak_) and HR (HR_peak_) values were measured during the 6MWT. The average VO_2_ (VO_2avg_) and HR (HR_avg_) values for the entire 6MWT were calculated. Cardiorespiratory measurements were not assessed separately for each movement of walking, turning or rest during the 6MWT.

### 2.4. Data Analysis

#### 2.4.1. Exercise Intensity and Walking Efficiency

First, exercise intensity was evaluated using the metabolic equivalent of tasks (METs) and the Karvonen method [26]. We set 1 MET as 2.7 mL/kg/min for participants with SCI [27] and classified the exercise intensity based on the METs into low (<3 METs), moderate (3–6 METs), and vigorous (>6 METs) levels [27,28]. The highest METs (MET_peak_) and average METs (MET_avg_) during the 6MWT were calculated. Second, exercise intensity was also determined using the Karvonen method, with HR relative to the HR reserve (%HRR) [26,28]. The %HRR was calculated as the difference between HR_rest_ and the age-predicted maximal HR, as shown in Equation (1). Exercise intensity according to the %HRR was categorized into light (30–39%), moderate (40–59%), and vigorous (60–89%) levels [8,26,28]. Third, walking efficiency was estimated using the oxygen cost of walking (O_2walking_), calculated as VO_2_ divided by walking speed (m/min) [17]. O_2walking_ refers to the amount of oxygen consumed per 1 kg of body weight to walk a 1-m distance.

(1) $$\%\mathrm{HRR}\;=\frac{\left(\mathrm{current}\;\mathrm{HR}\;-{\;\mathrm{HR}}_{rest}\right)}{\left(\mathrm{age}\;\mathrm{predicted}\;\mathrm{maximal}\;\mathrm{HR}\;-{\;\mathrm{HR}}_{rest}\right)}\times 100=\frac{\left(\mathrm{current}\;\mathrm{HR}\;-{\;\mathrm{HR}}_{rest}\right)}{\left(\left(220-\;\mathrm{age}\right)-{\;\mathrm{HR}}_{rest}\right)}\times 100,$$

#### 2.4.2. Statistical Analysis

Friedman tests were conducted to compare the changes of outcome variables at pre-, mid-, and post-training. A *p*-value of <0.05 was considered statistically significant. After the Friedman test, post-hoc analysis was performed using a Wilcoxon signed rank test. The Bonferroni correction was used by adjusting the *p*-value for multiple comparisons (*p* < 0.05/3). Statistical analyses were performed using SPSS software version 24 (IBM/SPSS Inc., Armonk, NY, USA).

## 3. Results

### 3.1. Participants

Of the 19 participants who were screened, one was excluded due to insufficient postural stability, and another seven participants did not want to engage in the study for personal reasons such as scheduling conflicts or difficulty coming to the laboratory. Eleven participants were enrolled in this study, and one participant withdrew before the initial training session because of scheduling conflicts. A total of 10 participants completed the training sessions. The demographic characteristics and clinical information of the 10 participants are presented in Table 1. The participants included seven men and three women with age ranging from 35 to 63 years (mean ± standard deviation, 48.1 ± 8.7) and time since injury ranged from 1.1 to 15.6 years (mean ± standard deviation, 5.7 ± 4.8). All participants had intact upper-extremity motor function except for one participant with a C6 neurologic level of injury. However, this latter participant had sufficient upper-extremity strength (all upper-extremity muscle grade ≥ 4 out of 5) to conduct walking training with an exoskeleton.

### 3.2. Walking Distance

The results of the 6MWT and cardiorespiratory measurements are presented in Table 2. In the 6MWT, the participants walked a significantly further distance at mid-training (37.5 ± 10.5 m) than at pre-training (20.7 ± 5.5 m) (*p* = 0.005) and covered more distance at post-training (49.1 ± 15.2 m) than at pre- and mid-training (*p* = 0.05 and *p* = 0.014, respectively).

### 3.3. Cardiorespiratory Outcomes

VO_2rest_, VO_2peak_, and VO_2avg_ were not significantly different after walking training. HRavg during the 6MWT at post-training (100.3 ± 10.7) decreased compared with that at pre-training (111.5 ± 17.2), at a borderline significance level (*p* = 0.028) and was significantly decreased compared with that at mid-training (110.0 ± 11.7) (*p* = 0.009).

### 3.4. Exercise Intensity and Walking Efficiency

**Figure 2:**
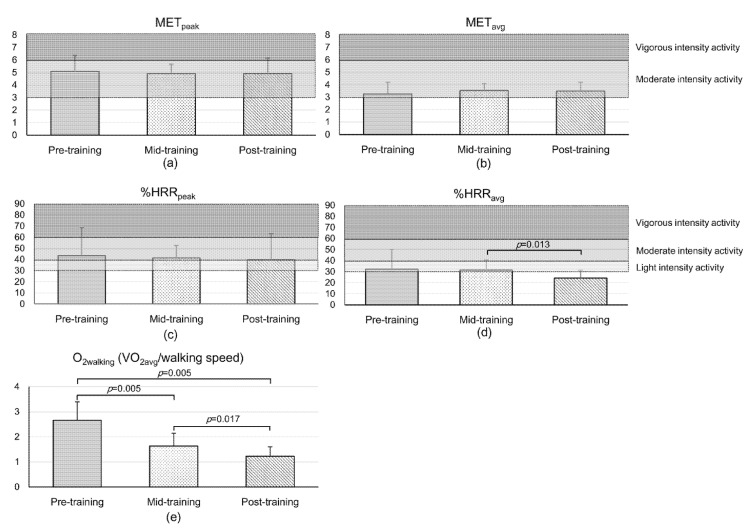
Changes in exercise intensity measured according to the (**a**) peak and (**b**) average (avg) metabolic equivalent of tasks (METs) for persons with spinal cord injury, (**c**) peak and (**d**) average heart rate relative to the heart rate reserve (%HRR), and (**e**) O_2_ cost of walking measured using oxygen consumption (VO_2avg_ in mL/kg/min) divided by walking speed (m/min) (O_2walking_).

The exercise intensity calculated according to cardiorespiratory outcomes and walking efficiency is provided in Figure 2. The categorization of exercise intensity is indicated by gray shading. The MET_peak_ during the 6MWT at pre-, mid-, and post-training was 5.1 ± 1.3, 4.9 ± 0.7, and 4.9 ± 1.1, respectively (pre- vs. mid-, *p* = 0.959; pre- vs. post-, *p* = 0.799; mid- vs. post- *p* = 0.799), and the MET_avg_ was 3.3 ± 0.9 at pre-training, 3.5 ± 0.6 at mid-training, and 3.5 ± 0.7 at post-training (pre- vs. mid-, *p* = 0.285; pre- vs. post-, *p* = 0.445; mid- vs. post-, *p* = 0.646). There was no difference in the MET_peak_ or MET_avg_ even after exercise.

The %HRR_peak_ changed from 43.4 ± 25.4% (pre-training) to 41.3 ± 11.1% (mid-training) to 39.6 ± 23.6 (post-training) (pre- vs. mid-, *p* = 0.959; pre- vs. post-, *p* = 0.575; mid- vs. post-, *p* = 0.074), and the %HRR_avg_ changed from 32.2 ± 17.9% (pre-training) to 31.6 ± 8.9% (mid-training) to 24.3 ± 7.3% (post-training) (pre- vs. mid-, *p* = 0.799; pre- vs. post-, *p* = 0.114; mid- vs. post-, *p* = 0.013). O_2walking_ significantly decreased from 2.7 ± 0.7 at pre-training to 1.6 ± 0.5 at mid-training to 1.2 ± 0.4 at post-training (pre- vs. mid-, *p* = 0.005; pre- vs. post-, *p* = 0.005; mid- vs. post-, *p* = 0.017).

## 4. Discussion

Our results demonstrated that the exercise intensity of walking with a robotic exoskeleton according to the METs (both peak and average) corresponded to a moderate level and did not change after 10 weeks of the walking training program, whereas the exercise intensity at pre-training according to the %HRR_peak_ and %HRR_avg_ corresponded to a moderate and light level, respectively, and decreased after 10 weeks of the walking training program. Furthermore, walking efficiency gradually improved after walking training with a robotic exoskeleton.

The cardiorespiratory responses assessed using VO_2_ in this study were consistent with those reported in previous studies, which indicated that the metabolic demand of walking with an exoskeleton is equivalent to a moderate level of physical activity [14,15,17,19]. The number of training sessions varied among the studies; however, exercise intensity was similar. In previous studies, the exercise intensity of exoskeleton-assisted walking was measured after a minimum of five training sessions [14], after approximately 40 training sessions [15], or after 4–14 training sessions (median, 10 sessions) [17]. Furthermore, a meta-analysis study by Miller et al. [10] demonstrated that, using a MET value of 2.7 mL/kg/min for participants with SCI, the average exercise intensity during exoskeleton-assisted walking was 3.3 METs, which is comparable to the MET_avg_ in this study. However, the exercise intensity evaluated according to the HR was moderate (%HRR_peak_) and less than moderate level (%HRR_avg_) in this study. Kozlowski et al. reported that exoskeleton-assisted walking corresponded to light to moderate exercise by estimating the METs using HR changes [16].

Several explanations could be proposed as to why the estimated exercise intensity of exoskeleton-assisted walking was different depending on the measurement methods. First, the reference value for estimating exercise intensity may be associated with these results. In this study, we used the reference value of the METs for patients with SCI (2.7 mL/kg/min), which is lower than that for healthy adults (3.5 mL/kg/min) [27]. Conversely, the %HRR was calculated using the age-predicted maximal HR (220-age), which was not adjusted for patients with SCI. In previous studies, the peak HR of patients with SCI was lower than the age-predicted maximal HR not only in patients with tetraplegia but also in patients with paraplegia [29,30]. Second, these results may be attributed to the innate characteristics of these measurement methods. The %HRR has been reported to have a tendency to underestimate exercise intensities, whereas the VO_2_ reserve tends to be overestimated in young healthy adults [31]. For these reasons, the exercise intensity calculated by %HRR may be underestimated compared to METs. Therefore, further research is needed to determine the reference value for %HRR in patients with SCI.

The results of the present study showed that VO_2_ did not changed, whereas HR_avg_ and %HRR_avg_ significantly decreased after 10 weeks of exoskeleton-assisted walking training. This is in agreement with the knowledge that regular aerobic exercise reduces the HR during submaximal workload due to decreased sympathetic drive and increased stroke volume, but does not change submaximal VO_2_ because of similar oxygen requirements for a fixed workload [32,33]. Although the activities of the trunk and upper extremity muscles were not evaluated in this study, it can be assumed that exoskeleton-assisted gait would be insufficient to induce muscle hypertrophy. Furthermore, despite the fact that exoskeleton-assisted walking training was not an overloading exercise, exoskeleton-assisted walking may be a tolerable aerobic exercise to induce cardiorespiratory responses considering the decrease in %HRR_avg_. In contrast, Kressler et al. reported no considerable changes in HR and VO_2_ during exoskeleton-assisted walking among three participants after six weeks of training [18]. These contradictory findings may have resulted from the duration of training, as other experimental designs including training methods, time, and frequency were similar between the previous study and this study. The duration of six weeks would be insufficient to make changes in cardiovascular activities, considering that no significant difference was observed in the HR or %HRR at week 5 in this study.

The energy cost for exoskeleton-assisted overground walking decreased after walking training. The progressive improvement in walking efficiency during the 10 weeks of training is a meaningful finding because faster exoskeleton-assisted walking may not require more metabolic demands as a result of walking proficiency. Further studies with larger sample sizes and training durations of >10 weeks are needed to identify the effects of walking training on walking efficiency and cardiorespiratory responses.

This study had some limitations. First, the study population was small and relatively heterogeneous, limiting the generalizability of the results. Second, cardiopulmonary exercise tests for evaluating maximal VO_2_ and HR were not conducted. Third, it was not possible to identify the effects of exoskeleton-assisted walking training for more than 10 weeks. Fourth, rest time during the 6MWT was not recorded, and cardiorespiratory responses during turn and rest were not assessed separately. Thus, the effects of rest and turning cannot be demonstrated. Finally, we did not assess how long the effects of exoskeleton-assisted walking for 10 weeks lasted after training.

## 5. Conclusions

Exoskeleton-assisted overground walking in patients with chronic SCI is generally compatible with a moderate level of physical activity. Regular walking training using an exoskeleton for 10 weeks progressively improves walking efficiency in chronic nonambulatory patients with SCI and may result in changes in cardiorespiratory responses.

## Figures and Tables

**Table 1:** Demographic and clinical characteristics of the participants.

No	Sex	Age (yrs)	Height (cm)	Weight (kg)	Time Since Injury (yrs)	NLI	AIS	L/E Motor Score
1	M	52	171	72.9	2.9	T10	A	0
2	M	46	180	75.6	4.8	T8	A	0
3	M	35	175	77.8	3.6	T10	A	0
4	F	58	163	75	7.9	C6	C	12
5	M	49	161	62	15.6	T10	A	0
6	M	49	167	74	3.5	T11	A	0
7	F	46	170	64.9	4	T10	A	2
8	F	35	160	52	12.1	L1	A	0
9	M	63	164	71.4	1.1	T4	C	15
10	M	48	172	70.1	1.3	T1	B	0
Mean ± SD		48.1 ± 8.7	168.3 ± 6.5	69.6 ± 7.9	5.7 ± 4.8			

Abbreviations: yrs = years, BMI = body mass index, NLI = neurologic level of injury, AIS = American Spinal Injury Association impairment scale, L/E = lower extremity, M = male, F = female, SD = standard deviation.

**Table 2:** Walking distance in the 6-min walk test and cardiorespiratory outcome measures.

	Pre-Training	Mid-Training	Post-Training	Pre- vs. Mid-	Pre- vs. Post-	Mid- vs. Post-
Total distance (m)	20.7 ± 5.5	37.5 ± 10.5	49.1 ± 15.2	0.005	0.005	0.014
VO_2rest_ (mL/kg/min)	3.6 ± 0.7	3.7 ± 1.0	3.2 ± 0.9	0.859	0.241	0.261
VO_2peak_ (mL/kg/min)	13.8 ± 3.4	13.2 ± 2.0	13.3 ± 3.3	0.999	0.799	0.838
VO_2avg_ (mL/kg/min)	8.8 ± 2.5	9.5 ± 1.5	9.4 ± 1.9	0.262	0.444	0.610
HR_rest_ (bpm)	84.0 ± 10.3	82.3 ± 9.0	77.7 ± 8.9	0.722	0.183	0.032
HR_peak_ (bpm)	121.1 ± 20.5	118.6 ± 13.6	115.7 ± 27.7	0.609	0.284	0.074
HR_avg_ (bpm)	111.5 ± 17.2	110.0 ± 11.7	100.3 ± 10.7	0.721	0.028	0.009

Abbreviations: VO_2_ = oxygen consumption, avg = average, HR = heart rate, bpm = beat per minute; Values are mean ± standard deviation.

## Data Availability

The data presented in the study are available upon request from the corresponding author. The data are not publicly available because of reasons concerning the privacy of the participants.
